# Integrin and autocrine IGF2 pathways control fasting insulin secretion in β-cells

**DOI:** 10.1074/jbc.RA120.012957

**Published:** 2021-01-13

**Authors:** Caroline Arous, Maria Luisa Mizgier, Katharina Rickenbach, Michel Pinget, Karim Bouzakri, Bernhard Wehrle-Haller

**Affiliations:** 1 Department of Cell Physiology and Metabolism, Centre Médical Universitaire, University of Geneva, Geneva, Switzerland; 2 UMR DIATHEC, Centre Européen d'Etude du Diabète, UMR DIATHEC, Strasbourg, France

**Keywords:** insulin secretion, integrin, insulin/insulin-like growth factor 1 (IGF1)-receptor signaling, IGF2, AKT isoform, beta cell (B-cell), insulin-like growth factor (IGF), insulin, Akt PKB, IGF1 receptor signaling, insulin receptor signaling

## Abstract

Elevated levels of fasting insulin release and insufficient glucose-stimulated insulin secretion (GSIS) are hallmarks of diabetes. Studies have established cross-talk between integrin signaling and insulin activity, but more details of how integrin-dependent signaling impacts the pathophysiology of diabetes are needed. Here, we dissected integrin-dependent signaling pathways involved in the regulation of insulin secretion in β-cells and studied their link to the still debated autocrine regulation of insulin secretion by insulin/insulin-like growth factor (IGF) 2–AKT signaling. We observed for the first time a cooperation between different AKT isoforms and focal adhesion kinase (FAK)–dependent adhesion signaling, which either controlled GSIS or prevented insulin secretion under fasting conditions. Indeed, β-cells form integrin-containing adhesions, which provide anchorage to the pancreatic extracellular matrix and are the origin of intracellular signaling via FAK and paxillin. Under low-glucose conditions, β-cells adopt a starved adhesion phenotype consisting of actin stress fibers and large peripheral focal adhesion. In contrast, glucose stimulation induces cell spreading, actin remodeling, and point-like adhesions that contain phospho-FAK and phosphopaxillin, located in small protrusions. Rat primary β-cells and mouse insulinomas showed an adhesion remodeling during GSIS resulting from autocrine insulin/IGF2 and AKT1 signaling. However, under starving conditions, the maintenance of stress fibers and the large adhesion phenotype required autocrine IGF2-IGF1 receptor signaling mediated by AKT2 and elevated FAK-kinase activity and ROCK-RhoA levels but low levels of paxillin phosphorylation. This starved adhesion phenotype prevented excessive insulin granule release to maintain low insulin secretion during fasting. Thus, deregulation of the IGF2 and adhesion-mediated signaling may explain dysfunctions observed in diabetes.

The regulated secretion of insulin from β-cells, residing in the islets of Langerhans of the pancreas, is critical for glucose homeostasis. In diabetic patients, elevated levels of basal insulin secretion under fasting conditions and insufficient glucose-stimulated insulin secretion (GSIS) after a meal represent the hallmarks of this pathology (1, 2) Although the molecular mechanisms involved in GSIS are well-understood, the control of the basal insulin secretion during starvation remains mysterious and requires further exploration. A complete comprehension of the signaling pathways involved in the control of insulin release under different physiologic conditions should provide new insights to understand the pathophysiology of diabetes.

Interestingly, mice exhibiting a β-cell–specific deletion of the insulin receptor (IR) or insulin growth factor 1 receptor (IGF1R) are glucose-intolerant and show fasting hyperinsulinemia (3, 4, 5). Similarly, a reduction in the activity of the serine–threonine kinase AKT induced defects in GSIS, and an increase of basal insulin levels in 6–8-week-old mice (6). However, the physiological relevance of an *in vivo* autocrine signaling effect of insulin is difficult to analyze, and several studies reported opposite results (7). Insulin downstream signaling elements such as phosphoinositide 3-kinase (PI3K) (8), protein kinase B (PKB/AKT) (6), or the small GTPase AKT substrate 160 (AS160) (9) have been shown to contribute to β-cell functions and GSIS (10). However, earlier experiments demonstrated a reduction of GSIS in the presence of exogenous insulin (11, 12, 13). Paradoxically, insulin-stimulated insulin secretion was observed in isolated human β-cells (14) and in healthy human individuals (15). More recently, IGF2 was shown to be an important player in the autocrine regulation of β-cell function. IGF2 is secreted together with insulin and is involved in regulating β-cell mass and GSIS through IGF1R signaling (16, 17). These findings open the possibility of autocrine stimulation by insulin/IGF1 or IGF2 and suggest the involvement of IR and IGF1R and their downstream signaling in the regulation of both GSIS and basal insulin secretion in β-cells (18).

Interestingly, a cooperation between insulin/IGF signaling and integrin-mediated adhesions were reported in other cell types, such as Chinese hamster ovary cells, hepatocytes, or cancer cells (19, 20, 21). Currently, it is not clear whether such a mechanistic link exists in pancreatic β-cells and whether this link can be modulated by different levels of glucose in the medium.

The heterodimeric transmembrane receptors of β1-integrin provide the mechanical linkage between the pancreatic ECM and the cytoskeleton of β-cells, forming cell–matrix adhesions, also called focal adhesions (FAs). FAs regulate trafficking and exocytosis of insulin containing granules. Indeed, after glucose stimulation, the disruption of the actin cytoskeleton or remodeling of integrin-dependent adhesions induces a decrease of insulin granules at basal membrane, shown by total internal reflection fluorescence (TIRF) microscopy with utilization of NPY plasmid (to labeled insulin granules) correlated with a decrease of GSIS (22, 23, 24, 25).

In contrast, under low-glucose or starving conditions, only a few, large, peripheral, actin stress fiber–associated FAs are observed in β-cells (22, 23, 25). After glucose addition to these β-cells, FAs and the actin cytoskeleton are remodeled with a response time of 15–30 min corresponding to the second phase of glucose-induced insulin secretion.^3^ Following the rise of glucose, β-cells show enhanced spreading, F-actin reorganization into dense networks, and the accumulation of phosphorylated forms of FAK (p-Tyr^397^), paxillin (p-Tyr^118^), and ERK1/2 in newly formed small protrusions. All of these events are necessary for a full GSIS response (22, 26, 27). Similar to FAK, the proline-rich tyrosine kinase 2 (Pyk2) is also involved in cytoskeleton and adhesion regulation (28), but its role in the context of β-cells has not been properly studied. On the other hand, two Src family kinases (SFKs), c-Src and YES, were shown to control GSIS (27, 29).

Concerning the regulation of the actin cytoskeleton in β-cells, activation of the small GTPase RhoA and its downstream effector, Rho-associated kinase (ROCK), which favor stress fiber formation, have been shown to inhibit β-cell spreading and GSIS (30). We accordingly proposed that activation of ROCK is required to block insulin granule trafficking and exocytosis (25). On the other hand, the small GTPase, Rac1, known to be activated by integrin-dependent cell–matrix adhesions in fibroblasts (31), has been shown to mediate cytoskeletal remodeling in β-cells that promote the second phase of insulin secretion (32). However, the precise mechanisms by which FAs and the associated actin cytoskeleton are stimulated to control the regulated release of insulin have not been established yet.

Here, we explored a possible cooperation between autocrine insulin/IGF2 and FA-mediated signaling to control insulin secretion in response to changes in extracellular glucose levels in β-cells. We show for the first time the involvement of IR/IGF1R signaling in the regulation of both FA-mediated GSIS and restriction of basal insulin secretion, demonstrating a bifurcation of the signaling pathway at the levels of the AKT1 and AKT2 isoforms. Under high-glucose conditions, AKT1 stimulation by IR/IGF1R signaling induces GSIS. In contrast, under low-glucose conditions, IGF2–IGF1R–AKT2 signaling prevents insulin granule release by maintaining actin stress fibers and large FAs via stimulation of RhoA–ROCK activity in a FA and paxillin/FAK signaling–dependent manner. This concerted and synergistic activity is efficiently curbing the release of insulin granules under fasting conditions.

## Results

### IR/IGF1R inhibition affects insulin release differentially under basal or glucose-stimulated conditions

**Figure 1:**
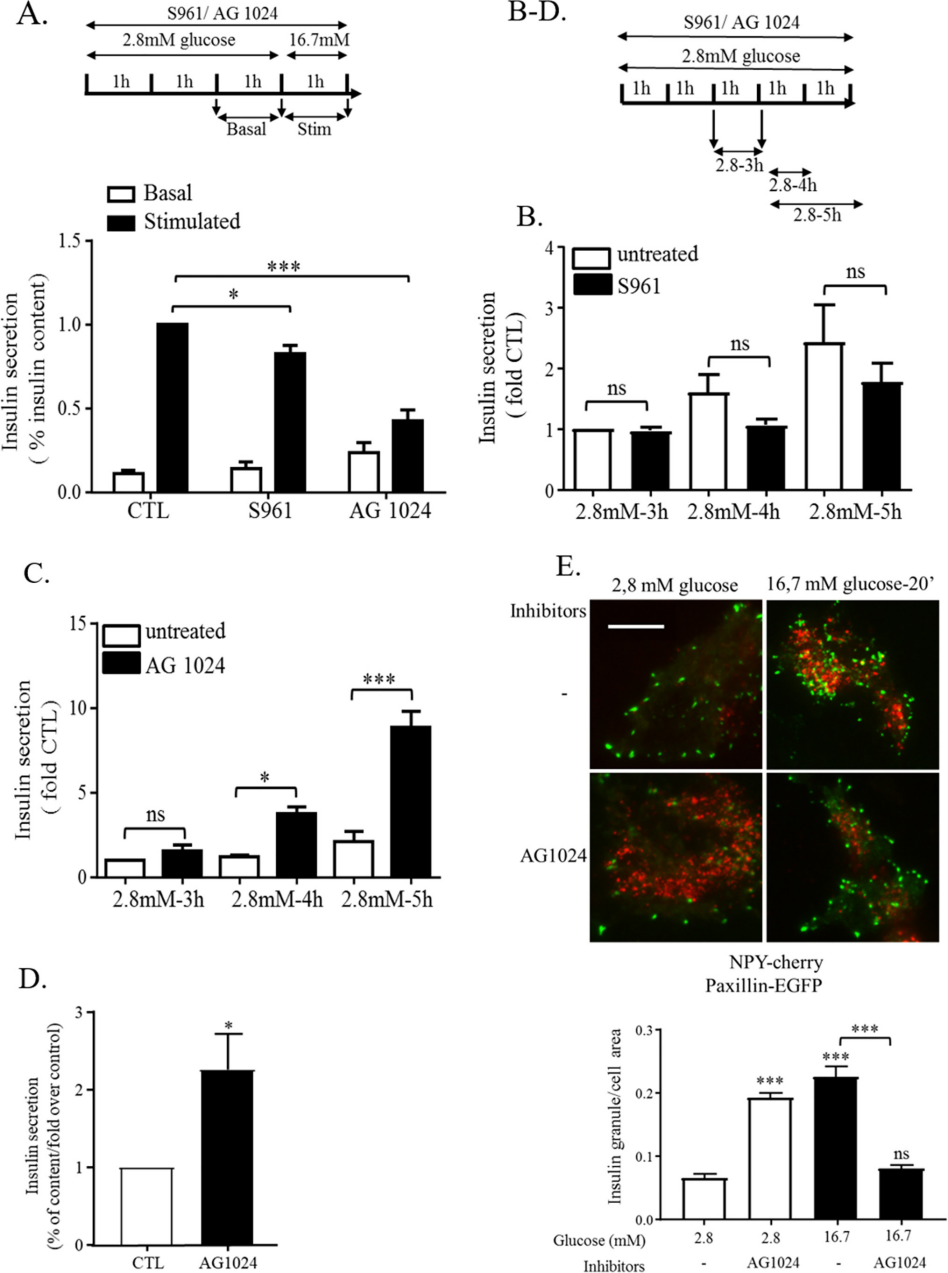
**IR/IGF1R inhibition by AG1024 affects insulin release differentially under basal (starving) and glucose-stimulated conditions in rat primary β-cells and islets.***A*, rat primary β-cells were incubated in 2.8 mm glucose medium for 2 h alone (*CTL*) or in the presence of the inhibitors for either the insulin receptor (S961, 0.1 μm) or insulin and IGF1 receptors (AG1024, 2.5 μm). Then cells were incubated 1 h more in low glucose ± inhibitors (basal, 2.8 mm, *white bars*) and stimulated with high glucose for 1 h (stimulated, 16.7 mm, *black bars*) in the continued presence or absence of the corresponding inhibitors (*n* = 7 independent experiments). Basal and GSIS were measured by radioimmunoassay and ELISA. *B* and *C*, rat primary β-cells were cultured in 2.8 mm glucose for 3, 4, or 5 h in control medium (untreated, *white bars*) or in the presence of S961 (*B*, *black bars*) (*n* = 7) or AG1024 (*C*, *black bars*) (*n* = 4). *D*, insulin release of rat whole islets cultured in 2.8 mm glucose in the presence or absence of AG1024 for 5 h (*n* = 4). *E*, MIN6B1 cells plated on glass-bottomed dishes were transfected with NPY–Cherry (to label insulin granule) and paxillin–EGFP plasmids and cultured for 72 h in growth medium. The cells were then incubated for 4 h in 2.8 mm glucose medium in the presence or absence (*CTL*) of AG1024. The cells were then fixed and analyzed by TIRF microscopy. The insulin granules present at basal membranes were counted and reported in respect to cell area (*n* = 4). Images are representative of four independent experiments. *Scale bars*, 10 μm.

To show a potential role for autocrine signaling of the insulin/IGF1 receptors pathway on insulin release in primary rat β-cells, we first determined whether treatment with inhibitors of this signaling pathway could affect insulin secretion. S961, a specific insulin receptor antagonist (33), and AG1024, a dual IGF1R and IR kinase inhibitor (34), were used to block potential autocrine signaling events. Interestingly, both S961 and AG1024 reduced GSIS without significantly affecting basal insulin secretion under low-glucose conditions (Fig. 1*A*). Nevertheless, we observed a tendency of basal insulin secretion to increase when β-cells were treated for 3 h with AG1024. To determine whether longer inhibitor treatment would affect the basal levels of insulin release in low glucose, the treatments were prolonged to 4 and 5 h before measuring insulin released during a 1-h interval. Interestingly, AG1024 but not S961 induced a significant increase of basal insulin secretion after 4 and 5 h of treatment of rat primary β-cells under low-glucose conditions (Fig. 1, *B* and *C*) without affecting cell survival (Fig. S1). Accordingly, similar effects were observed on whole rat islets, where AG1024 increased insulin secretion under low-glucose conditions (Fig. 1*D*). However, after glucose stimulation for 20 min in the presence of AG1024, the amount of insulin granules localized at the basal membrane dropped (Fig. 1*E*), potentially reflecting a reduced release of insulin in the second phase of GSIS.

This differential insulin secretion response in primary rat β-cells was paralleled in mouse MIN6B1 cells by analyzing the recruitment of insulin granules to the basal membranes by TIRF microscopy, which has been shown to reflect insulin secretion levels (23, 25, 35). Importantly, mouse β-cell–derived MIN6B1 cells used for this experiment demonstrate similar FA and actin remodeling, as well as glucose-induced insulin secretion, when compared with rat primary β-cells (23). In low-glucose conditions, treatment with AG1024 enhanced the presence of insulin granules at the basal membrane, which correlated with the increased release of basal insulin secretion under starving conditions in the presence of this drug (Fig. 1*C*). In contrast, after glucose stimulation for 20 min in the presence of AG1024, fewer insulin granules localized at the basal membrane (Fig. 1*E*), potentially reflecting a reduced release of insulin during the second phase of GSIS. Therefore, our data propose that (i) IR/IGF1R signaling is required to maintain a low level of basal insulin secretion under low-glucose conditions, and (ii) the IR/IGF1R signaling is required to produce an efficient insulin release in the second phase of GSIS. To better characterize the different signaling pathways involved in GSIS or granule retention under low glucose, respectively, we decided to further explore how modulation of the IR/IGF1R pathway affects β-cell morphology, previously linked to insulin granule secretion.

### IR/IGF1R inhibition affects glucose-mediated actin and FA remodeling

**Figure 2:**
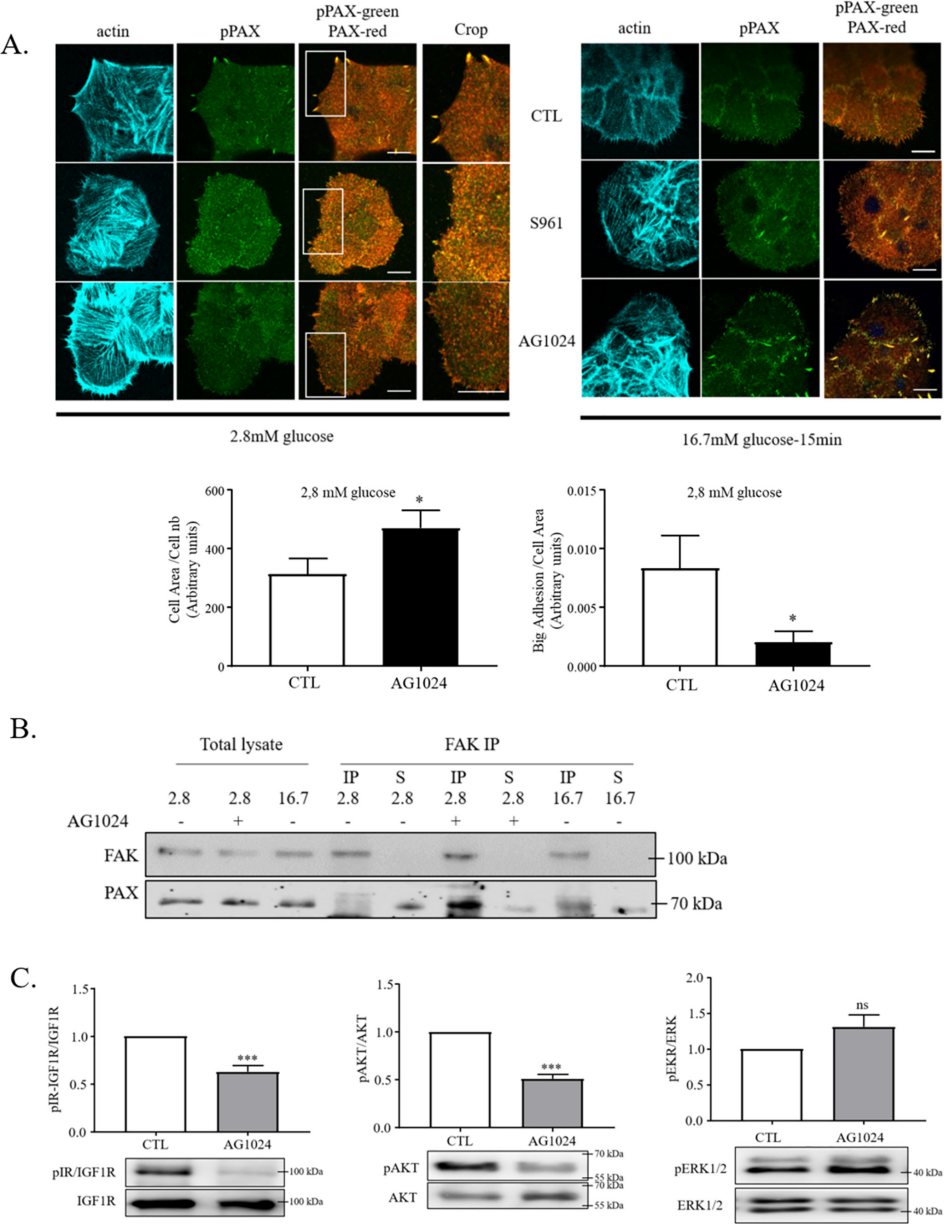
**IR/IGF1R signaling regulates basal and glucose-stimulating insulin secretion through adhesion remodeling.***A*, rat primary β-cells were cultured in 2.8 mm glucose medium for 4 h without inhibitors (*CTL*) or with S961 or AG1024 (some dishes were fixed corresponding to the 2.8 mm glucose condition) prior to stimulation with glucose (16.7 mm) for 20 min in the presence or absence (*CTL*) of S961 or AG1024. The cells were then fixed and stained for phospho-Tyr^118^–paxillin (*pPAX* in *green*), paxillin (*PAX-red*), or F-actin with Alexa 647–conjugated phalloidin (*cyan*). Cell area and number of big adhesions were counted for the condition of 2.8 mm glucose in the presence or absence of AG1024. (We defined a big adhesion as an adhesion with its length and width larger than 2 and 0.5 μm, respectively (*n* = 6). *Scale bars*, 10 μm. *B*, rat primary β-cells were incubated in 2.8 mm glucose for 4 h alone or treated with AG1024 and then lysed or followed by stimulation in 16.7 mm glucose-containing medium for 20 min. Cell lysates were immunoprecipitated with anti-FAK antibodies. Then total cell lysates, immunoprecipitations (*IP*), and immunoprecipitation supernatants (*S*) were run on SDS-PAGE and analyzed by Western blotting. The presented blot is representative of four independent experiments. *C*, MIN6B1 cells were treated with AG1024 or not (*CTL*) for 4 h in the presence of 2.8 mm glucose. Representative Western blots stained against the respective proteins were quantified. The ratio of phospho-IR–IGF1R and total IGF1R (*n* = 6), phospho-AKT and total AKT (*n* = 6), and phospho-ERK1/2 and total ERK 1/2 (*n* = 7) were performed for each condition and compared against 2.8 mm glucose condition without inhibitors (fold over CTL).

In agreement with previous studies (22), incubating β-cells at 2.8 mm glucose concentration induced the formation of big and large FAs connected by F-actin–rich stress fibers. The subsequent addition of 16.7 mm glucose for 20 min induced insulin secretion but also enhanced cell spreading, and the formation of numerous small peripheral protrusions containing F-actin and integrin-associated p-Tyr^118^ phosphorylated paxillin. Thus GSIS is associated with F-actin and FA remodeling, which has been shown to be important for the second phase of insulin secretion (24). Because the autocrine signaling of IR and IGF1R was relevant for GSIS, we asked whether their signaling was also required for F-actin and FA remodeling under low or high glucose concentrations. In the absence of inhibitors, β-cells cultured under low-glucose conditions displayed large FAs and stress fibers. These structures were remodeled after glucose addition into small F-actin protrusions, which contained newly formed cell–matrix adhesions positive for phosphopaxillin (Fig. 2*A*). Surprisingly, similar phenotypes were observed when IR and IGF1R signaling were blocked by either S961 or AG1024 under low-glucose concentration, resulting in the formation of F-actin–rich protrusions and many small adhesions at the cell periphery comparable with the stimulated condition (Fig. 2*A*). Likewise, AG1024 treatment under low-glucose enhanced cell spreading and basal insulin secretion (Fig. 1, *C* and *D*). After 20 min of glucose stimulation, inhibitor- treated cells showed large and small adhesions phenotypes (Fig. 2*A*), suggesting that IR and IGF1R signaling are involved in GSIS regulation but only partially through adhesion remodeling.

The association of paxillin with FAK and their co-recruitment to nascent peripheral adhesions was shown to depend on paxillin phosphorylation at residues Tyr^31^ and Tyr^118^ in fibroblast (36). Here, in rat β-cells, co-immunoprecipitation of paxillin with anti-FAK antibodies was barely detectable after 4 h in low glucose, but increased after 20 min of high glucose (Fig. 2*B*). AG1024 treatment under low-glucose conditions also showed the FAK/paxillin association by co-immunoprecipitation (Fig. 2*B*), which correlated with the formation of many peripheral paxillin reactive adhesions and an increase in F-actin (Fig. 2*A*). This suggests that under low-glucose conditions, IGF1R signaling induces few stress fiber–linked large focal adhesions, in which the paxillin/FAK association is reduced with the apparent goal to maintain low levels of insulin secretion. To confirm that IR-IGF1R signaling is effectively blocked by AG1024, we analyzed the phosphorylation status of insulin and IGF1 receptors and their downstream targets AKT and ERK in low-glucose conditions. Both receptors and AKT phosphorylation were decreased in the presence of the inhibitors, but ERK phosphorylation was unchanged, potentially reflecting a compensating integrin-mediated signaling (Fig. 2*C*). To establish a direct link between the IR/IGF1R–PI3K–AKT signaling pathway and the FA state under high- and low-glucose conditions, we treated cells with the PI3K inhibitor (LY294002). Similar to AG1024, inhibition of PI3K with LY294002 enhanced the formation of paxillin-containing peripheral adhesions in low-glucose conditions (Fig. S2), suggesting that an autocrine insulin/IGF2 signaling modulates FA adhesions in low-glucose conditions.

### Autocrine IGF2 prevents impaired insulin secretion and FA remodeling under low-glucose condition

**Figure 3:**
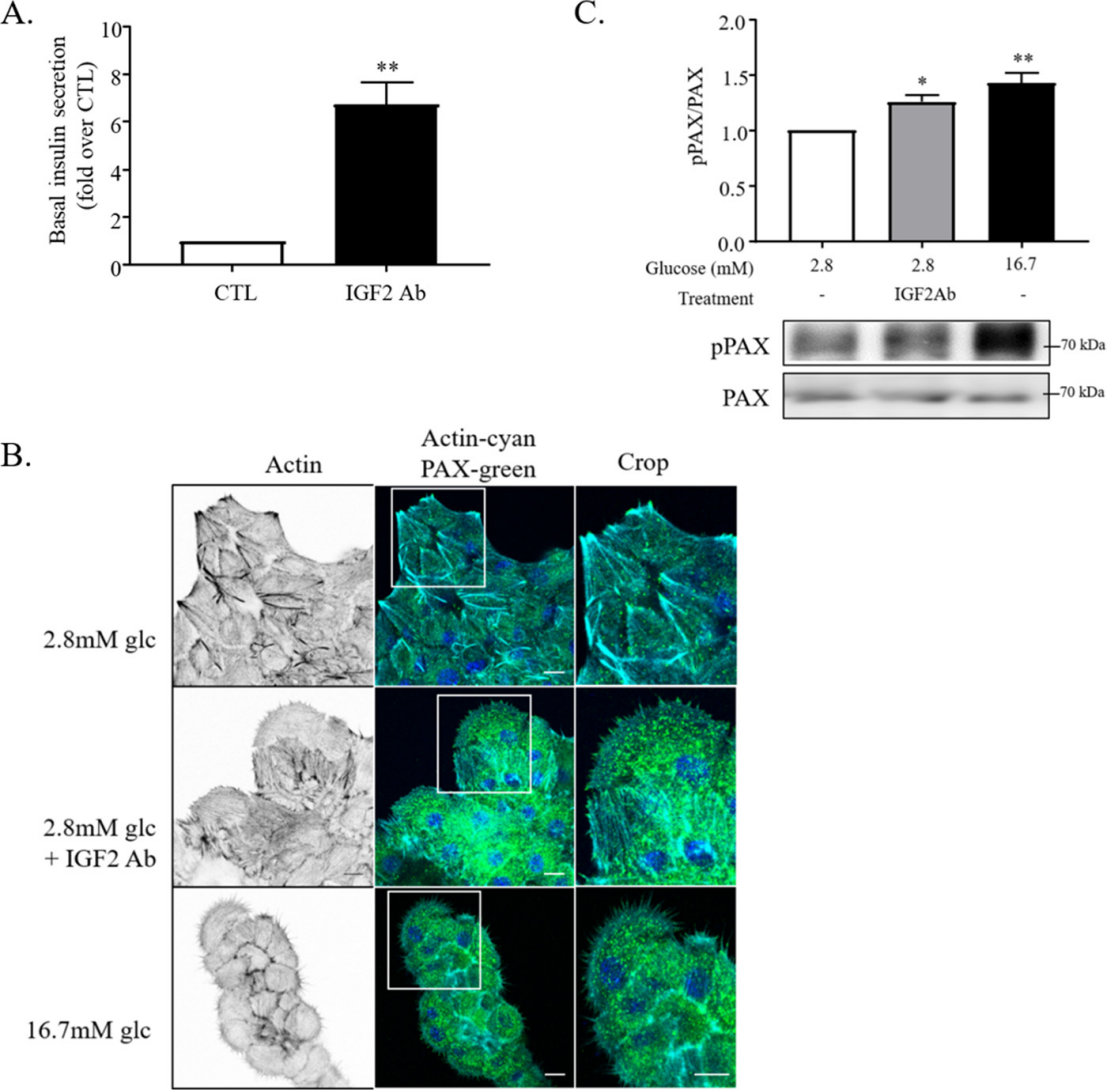
**Low basal insulin secretion is regulated by secreted IGF2 to maintain a large adhesion phenotype.***A* and *B*, rat primary β-cells were cultured in 2.8 mm glucose medium for 5 h, containing anti-IGF2 blocking antibody (IGF2ab, 0.1 µg/ml) or not (*CTL*). *A*, insulin secretion was then measured by ELISA (*n* = 3). *B*, cells were subsequently fixed and stained for paxillin (*green*) and F-actin Alexa 647–conjugated phalloidin (*cyan*). Images are representative of three independent experiments. *C*, MIN6B1 cells were cultured in 2.8 mm glucose medium for 5 h in the presence or absence of anti-IGF2 blocking antibodies and stimulated for 20 min with 16.7 mm glucose medium. Representative Western blotting and quantification of phospho-Tyr^118^–paxillin and total paxillin were performed and compared against 2.8 mm glucose condition without inhibitors (fold over *CTL*, *n* = 4). *Scale bars*, 10 μm.

Based on our experiments with the IR/IGF1R inhibitor AG1024, we hypothesized that autocrine IGF2-mediated signaling could restrict basal insulin secretion under low-glucose conditions. Indeed, IGF2 was shown to be secreted concomitantly with insulin during granule exocytosis and regulated insulin secretion in an autocrine manner (17, 37). IGF2 and insulin bind both to IGF1R and IR, however with different affinities (38). To validate our hypothesis, IGF2-blocking antibodies were applied to rat primary β-cells in low-glucose conditions, which resulted in an increase in basal insulin secretion (Fig. 3*A*) and the concomitant remodeling of F-actin and the formation of small paxillin-enriched nascent adhesions (Fig. 3*B*). In addition, we observed an increase in paxillin Tyr phosphorylation induced by IGF2 blockade by Western blotting (Fig. 3*C*), which further supported the nascent adhesion phenotype of primary β-cells, normally induced by 20 min in high glucose. Thus, our data confirm the notion that released IGF2 activates the IGF1R–PI3K signaling pathway in an autocrine fashion, which induces the formation of stress fibers and large FAs, to restrict insulin release in low-glucose condition.

### Unique function of AKT isoforms: AKT1 is required for GSIS, whereas AKT2 prevents fasting insulin release by stimulating the formation of stress fibers and large FAs

**Figure 4:**
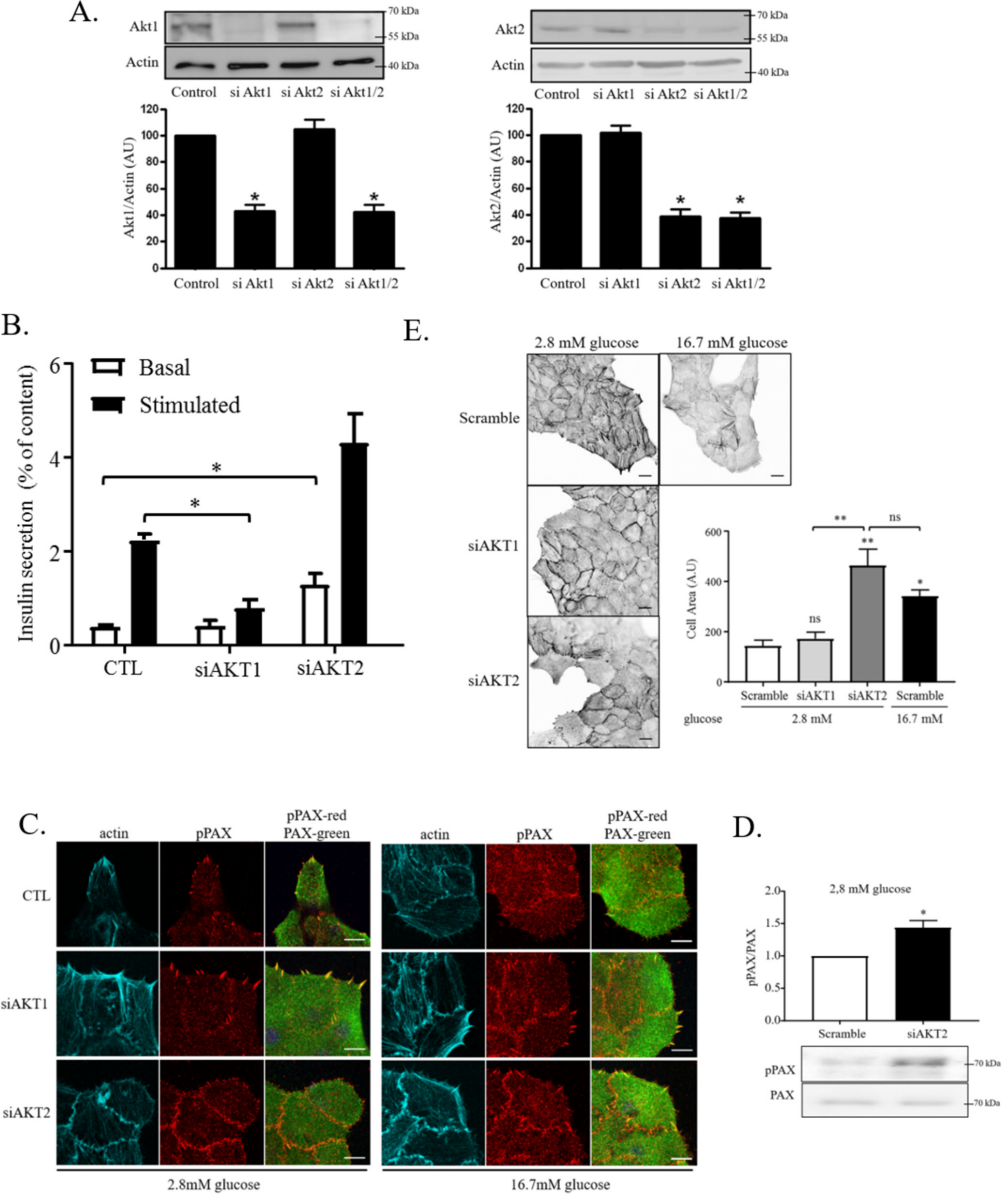
**AKT1 is involved in GSIS, whereas AKT2 prevents basal insulin release by inhibiting adhesion remodeling, cell spreading, and paxillin phosphorylation.***A*, AKT1 or AKT2 isoforms or scramble (control) specific siRNA were transfected into rat primary β-cells and incubated for 72 h and analyzed by Western blotting for expression of AKT-variants. *B*, after 72 h of transfection, the cells were preincubated 2 h at 2.8 mm glucose and then incubated for 1 h in 2.8 mm glucose (*white bars*) followed by 1 h at 16.7 mm glucose (*black bars*), and insulin secretion was measured by radioimmunoassay and compared with total insulin content in cells. *C*, transfected β-cells were cultured in 2.8 mm glucose for 4 h and then stimulated or not with glucose (16.7 mm) for 20 min. The cells were subsequently fixed and stained for phosphopaxillin (*red*), paxillin (*green*), and F-actin (Alexa 647–conjugated phalloidin, *cyan*). The images are representative of three independent experiments. *Scale bars*, 10 μm. *D*, representative Western blotting and quantification of phospho-Tyr^118^–paxillin and total paxillin in primary rat β-cells transfected with scrambled or AKT2 siRNA at 2.8 mm glucose for 4 h (*n* = 4). *E*, transfected cells were cultured in 2.8 mm glucose for 4 h and then stimulated with glucose (16.7 mm) for 20 min. The cells were then fixed and stained for F-actin to measure the cell area (*n* = 3). *Scale bars*, 10 μm.

Because IR/IGF1R signaling is involved in both GSIS at high, and maintenance of large FA/stress fibers under low-glucose conditions, we wondered at which level the PI3K/AKT signaling pathway could be differentially routed in a glucose-dependent manner. The serine/threonine protein kinase AKT consists of three different isoforms, all of which are expressed in human islets and β-cells (39, 40). Previous reports have demonstrated an isoform-specific role for AKT1 and AKT2 in the control of the cellular metabolism (41). To test the functions of the different AKT isoforms, we transfected primary β-cells with isoform-specific siRNAs. Transfection with selective siRNAs decreased AKT1 and AKT2 protein expression by 55 and 60%, respectively (Fig. 4*A*). Knockdown of AKT1 decreased GSIS in rat primary β-cells, whereas the suppression of AKT2 increased basal insulin secretion (Fig. 4*B*, *white bar*) and had no significant effect on GSIS (Fig. 4*B*, *black bars*). In respect to the glucose-mediated FA and actin remodeling, siAKT1 did not modify the formation of stress fiber–bound large FAs at low glucose but partially prevented their remodeling after addition of glucose to the medium (Fig. 4*C*). In addition, in low-glucose conditions, siAKT2-treated cells failed to form stress fibers and large FAs (Fig. 4*C*, *left panel*) but increased their paxillin phosphorylation (Fig. 4*D*) and cell area (Fig. 4*E*). Interestingly, the phenotype induced by knockdown of AKT2 in low glucose resembled glucose-induced changes in control β-cells. Taken together, our data show different functions of AKT1 and ATK2 isoforms in β-cells: (i) under low glucose, the IGF2–IGF1R–PI3K–AKT2 signaling axis restricts insulin secretion by maintaining actin stress fibers, large FA, and reduced Tyr–paxillin phosphorylation, and (ii) the IR/IGF1R–PI3K-AKT1 signaling is required for efficient GSIS and partially involved in glucose-induced remodeling of F-actin and FAs.

### FAK signaling restricts insulin secretion under low-glucose conditions

Because regulation of insulin release is linked to the morphology of paxillin-containing adhesions, which in turn is regulated by IGF2 signaling and levels of glucose in the medium, we next explored signaling molecules directly recruited to FAs. Previously, FAK and c-Src have been observed to play roles during GSIS and could therefore link adhesion remodeling with insulin granule release (22, 26, 27). Nevertheless, their specific function in regulating insulin secretion and their involvement in GSIS are not well-established. Therefore, the role of FAK and SFK signaling in FA and F-actin remodeling was explored by using kinase inhibitors for FAK/Pyk2 (PF562271) (42) and SFK family kinase, c-Src, YES, and Fyn (saracatinib or dasatinib) (43, 44).

**Figure 5:**
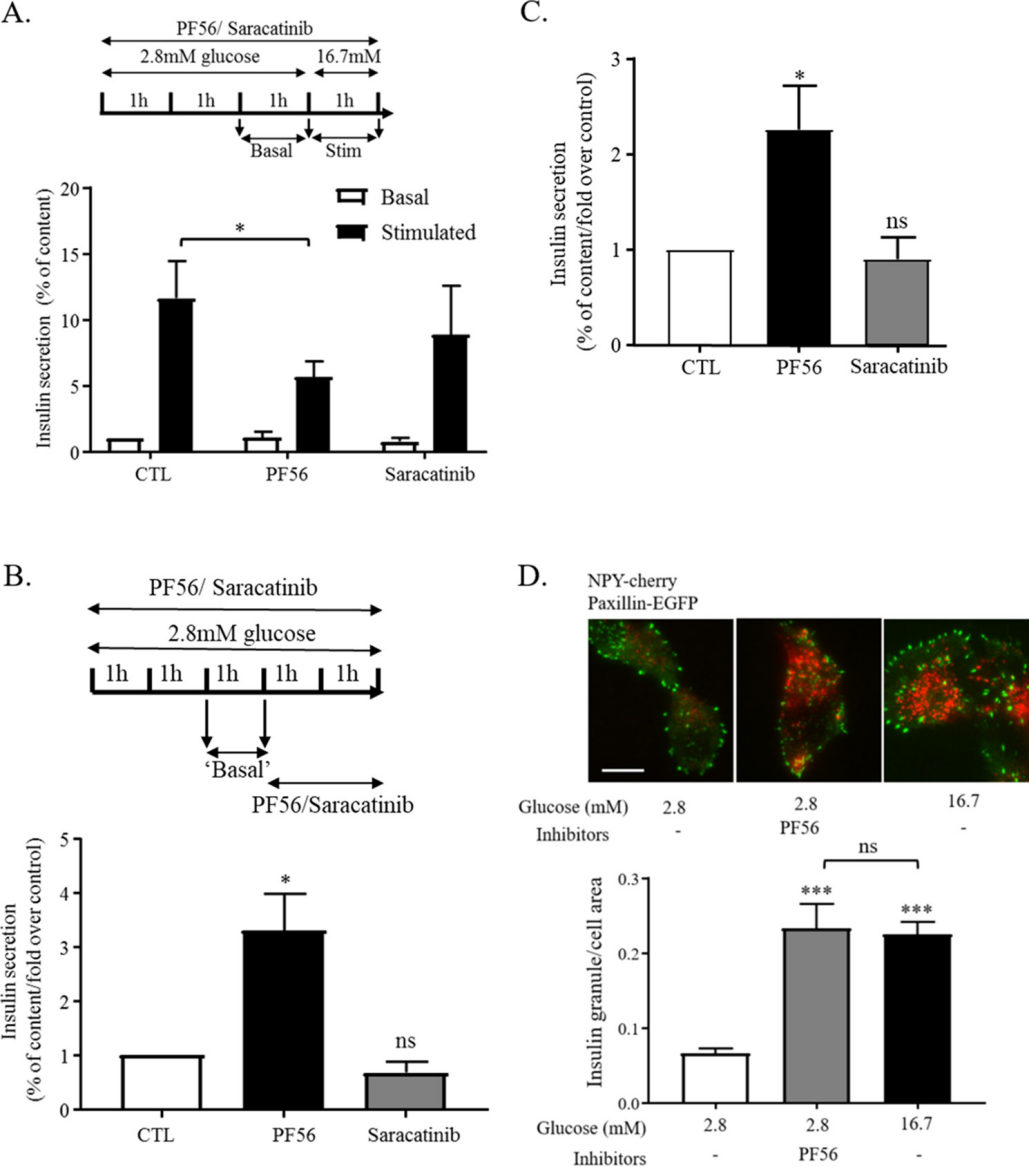
**FAK inhibition increases basal insulin secretion and insulin granules recruitment at basal membranes in rat primary β-cells and islets.***A*, rat primary β-cells were preincubated for 2 h with 2.8 mm glucose in the presence or absence (*CTL*) of the inhibitors PF562271 (*PF56*, 0.1 μm) and saracatinib (0.1 μm). Then cells were incubated 1 h more in low glucose ± inhibitors (basal, 2.8 mm, *white bars*) and stimulated with high glucose for 1 h (*Stim* or *Stimulated*, 16.7 mm, *black bars*) in the continued presence or absence of the corresponding inhibitors. Insulin secretion were measured by ELISA (*n* = 3). *B*, rat primary β-cells were cultured in 2.8 mm glucose for 5 h in the presence or absence (*CTL*) of PF562271 (*PF56*) or saracatinib. Insulin secretion were measured by ELISA (*n* = 3). *C*, experiment as in *B*, but whole rat islets were cultured in 2.8 mm glucose in the presence or absence of AG1024 for 5 h (*n* = 4). *D*, MIN6B1 cells were transfected with NPY–Cherry and paxillin–EGFP plasmids and cultured for 72 h in growth medium. The cells were then incubated in 2.8 mm glucose medium in the presence or absence (*CTL*) of AG1024 for 5 h. Then cells were fixed, and pictures were taken by TIRF microscopy. The insulin granules present at basal membrane were counted and reported to cell area (*n* = 4). *Scale bars*, 10 μm.

We first confirmed the inhibitory effect of saracatinib or dasatinib on glucose-induced phospho–c-Src remodeling in MIN6B1 cells by confocal microscopy (Fig. S3). In accordance with previous reports (22, 27, 29), blocking FAK/Pyk2 and SFK reduced GSIS (Fig. 5*A*). However, the inhibition of GSIS was significant only for the FAK/Pyk2 inhibitor (PF562271), and a tendency was observed with saracatinib (Fig. 5*A*, *black bars*). Neither inhibitor affected basal insulin release after 3 h. Because the AG1024-mediated increase in insulin release was observed after 4 h of glucose starvation, we also analyzed a potential effect of FAK/Pyk2 or SFK inhibition at this time point. Treating MIN6B1 cells or rat primary whole islets with PF562271 for 4 h under low-glucose conditions induced a significant increase in insulin release, which was, however, not observed with saracatinib (Fig. 5, *B* and *C*). Similar to treatment with AG1024 under low glucose, PF562271 induced the recruitment of insulin-containing granules to the basal plasma membrane under starvation, to levels comparable with that seen in GSIS (Fig. 5*D*).

**Figure 6:**
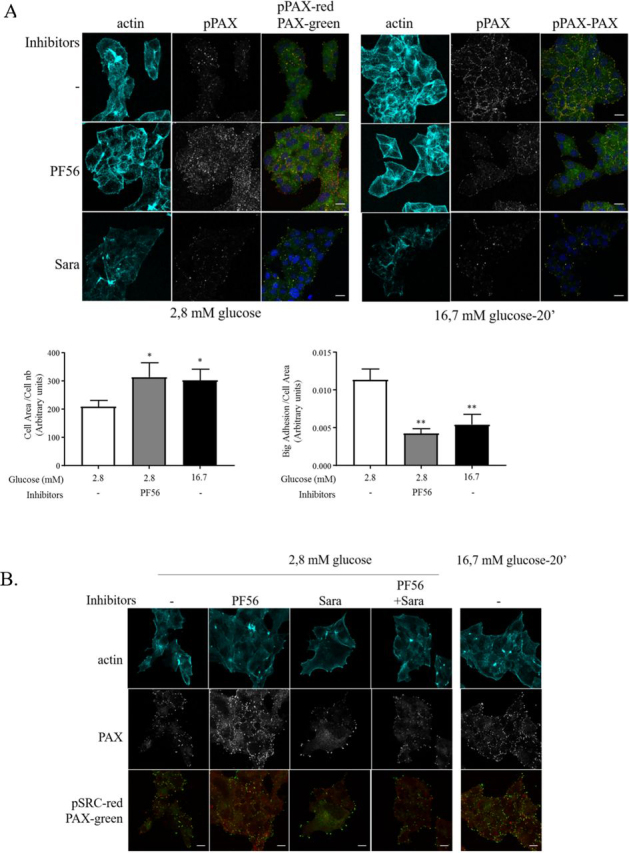
**FAK inhibition induces adhesion remodeling in low-glucose conditions through SFK activation.***A* and *B*, MIN6B1 cells were cultured in 2.8 mm glucose for 4 h in control (*CTL*) or inhibitor containing medium PF562271 (*PF56*, 0.1 μm) or saracatinib (*Sara*, 0.1 μm). The cells were then stimulated with 16.7 mm glucose for 20 min in the continued presence of the inhibitors. *A*, cells were subsequently fixed and stained for pPAX (*red*), paxillin (*green*), and F-actin (phalloidin in *cyan*). Cell area and number of big adhesions were counted for the indicated conditions 2.8 mm glucose ± PF562271 and high glucose (*n* = 6). *B*, cells were stained for phospho-Tyr^418^ c-Src (*pSRC* in *red*), paxillin (*green*), and actin (phalloidin in *cyan*). All images are fully representative of at least three independent experiments. *Scale bars*, 10 μm.

Because inhibition of FAK kinase activity would result in associated changes of c-Src and paxillin, we further analyzed F-actin and FA remodeling in MIN6B1 cells after 4 h in low glucose. Under this condition, inhibition of SFK family kinase by saracatinib did not affect adhesion remodeling, reflecting the situation observed for basal insulin secretion (Fig. 5, *B* and *C*). However, treating cells with the PF562271 inhibitor under low-glucose conditions increased the number of small p-Tyr^118^–paxillin reactive adhesions (Fig. 6*A*, *left panel*), which correlated with an increase in cell area and reduced numbers of large FAs (Fig. 6*A*). This phenotype correlated with an augmentation of basal insulin secretion (Fig. 5, *B* and *C*) and an increase of insulin granule recruitment at basal membranes (Fig. 5*D*). To establish the hierarchies between FAK and c-Src signaling, MIN6B1 cells were co-treated with PF562271 and saracatinib under low-glucose conditions, which reverted FA remodeling seen by FAK/Pyk2 inhibition (Fig. 6*B*), suggesting that the FAK inhibitor–induced remodeling process required SFK, potentially similar to the remodeling observed under high-glucose conditions. Thus, it appears that under low-glucose conditions, FAK/Pyk2 kinase but not SFK activity is required to maintain stress fibers and large FAs to control insulin secretion. Nevertheless, under high-glucose conditions, both activities are required for glucose-induced FA remodeling and insulin secretion as previously shown.

### Phosphopaxillin but not phospho-FAK controls FA remodeling in low glucose

**Figure 7:**
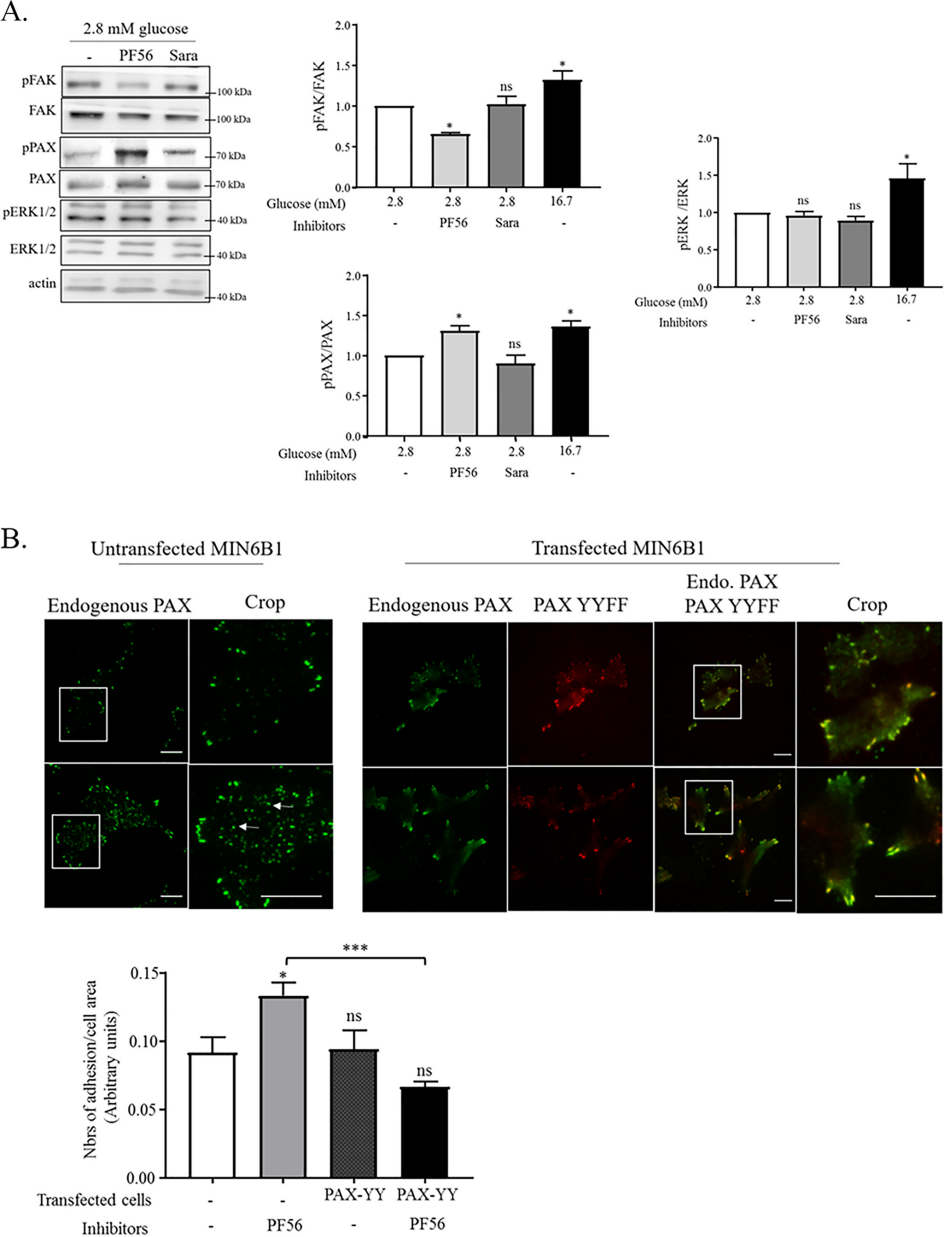
**FAK inhibition induces an increase of tyrosine phosphorylation of paxillin and adhesion remodeling in low-glucose condition.***A*, representative Western blotting and quantification of MIN6B1 cells treated for 4 h at 2.8 mm glucose in the presence or absence (*CTL*) of the inhibitors PF562271 (*PF56*) and saracatinib (*Sara*). The cells without inhibitors treatment were also stimulated for 20 min with 16.7 mm glucose. The ratios of phospho-Tyr^397^–FAK and total FAK (*n* = 4), phospho-Tyr^118^–paxillin and total paxillin (*n* = 5), and phospho-(T185/Y187) ERK1/2 and total ERK 1/2 (*n* = 4) were done for each condition and compared against 2.8 mm glucose condition without inhibitors (fold over control). *B*, MIN6B1 cells plated on glass-bottomed dishes were transfected (or not) with paxillin–Y31/118F–Cherry plasmid (*red*) and cultured for 72 h in normal medium. The cells were incubated with low glucose (2.8 mm) in the presence or absence of the PF562271 (*PF56*) for 4 h. The cells were subsequently fixed and stained for paxillin (PAX in *green*), and pictures were taken by TIRF microscope. The adhesions were counted and normalized with the cell area on more than 10 cells/condition (*n* = 3). *Scale bars*, 10 μm. *Endo.*, endogenous.

To better understand the differential glucose-mediated FAK activities, the dynamic association of paxillin with FAK and its phosphorylation was further explored. On the one hand, FAK phosphorylation on Tyr^397^ is necessary to recruit and activate c-Src at focal adhesion sites to form a FAK/c-Src signaling complex, in which c-Src is then proposed to induce the phosphorylation of Tyr^118^ of paxillin (45). On the other hand, p-Tyr^118^–paxillin shows enhanced FAK interaction in nascent adhesions (36), making it less clear which phosphorylation event occurs first. Under low-glucose conditions, the phosphoprotein analysis of MIN6B1 cells treated with either PF562271 or saracatinib inhibitors revealed a PF562271-dependent decrease in FAK phosphorylation on Tyr^397^, which was surprisingly associated with an increase of paxillin–Tyr^118^ phosphorylation (Fig. 7*A*). Although not consistent with some observations in the literature (36, 46, 47), this observation reflected the increase in phosphopaxillin observed by immunofluorescence of PF562271-treated MIN6B1 cells (Fig. 6*A*). In contrast, FAK inhibition had no effect on ERK phosphorylation, nor did c-Src inhibition modify the FAK, paxillin, and ERK phosphorylation status, confirming the absence of effects seen with saracatinib in low-glucose conditions (Fig. 7*A*). To confirm the crucial role of paxillin phosphorylation in regulation of FAs in low glucose, MIN6B1 cells were transfected with a Tyr phosphorylation–defective mutant of paxillin (paxillin-Y31F/Tyr^118^F-mCherry). Importantly, in the presence of the YYFF PAX mutant, FAK/Pyk2 inhibition (PF562271) failed to induce FA remodeling under low-glucose conditions, and the large FA phenotype was maintained, confirming that paxillin phosphorylation on Tyr^118^ is critical for FA remodeling when FAK/Pyk2-kinase activity is inhibited (Fig. 7*B*). This suggests that in the absence of phosphorylated Tyr^379^ FAK, SFK-driven paxillin phosphorylation may occur outside of focal adhesion (48), which then triggers F-actin and FA remodeling independent of FAK kinase activity, but subsequently triggering insulin granule release. Furthermore, by integrating our FAK pulldown data of AG1024-treated cells in low-glucose medium (Fig. 2*C*), the inhibition of the IR/IGF1R–AKT2 signaling increases the pool of activated phosphopaxillin, which may attract FAK to nascent adhesions in the cell periphery, but where it also triggers cell spreading and adhesion remodeling by potentially a Cdc42–PAK1–Rac1–mediated process (24, 35). However, normally, this signaling axis is prevented by FAK/Pyk2 signaling, which induces adhesion turnover and the formation of stress fibers and large FA-phenotype, preventing the release of insulin granules under starving conditions.

### RhoA/ROCK signaling prevents FA remodeling under low-glucose conditions

**Figure 8:**
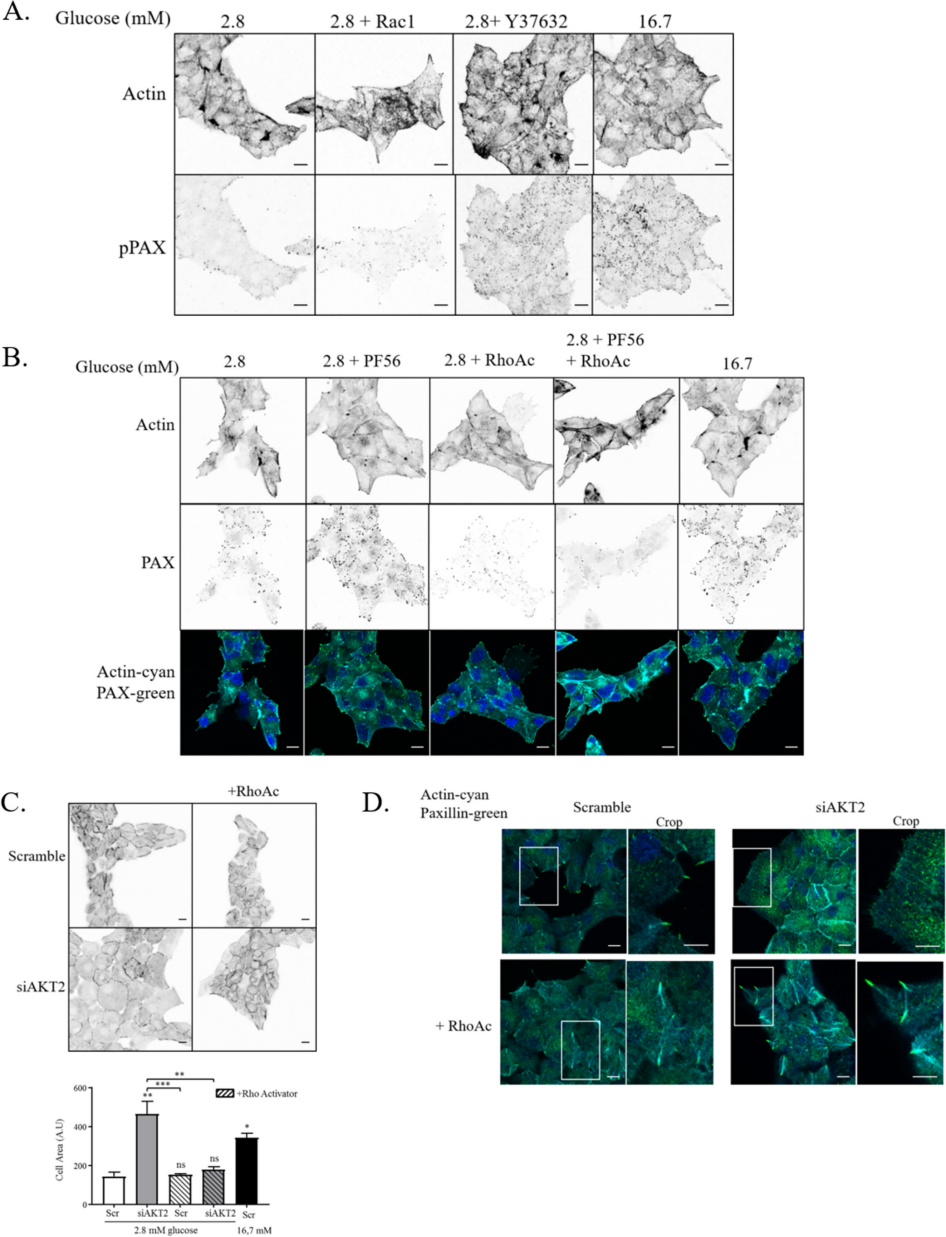
**Rho–ROCK signaling is necessary to maintain the big adhesion phenotype in low-glucose conditions and rescues FAK and AKT2 depletion.***A*, MIN6B1 were cultured in 2.8 mm glucose for 4 h in the presence or absence (*CTL*) of ROCK inhibitor (Y27632, 50 μm) or Rac1 inhibitor (0.2 μm) and then stimulated or not for 20 min with 16.7 mm glucose. The cells were subsequently fixed and stained for actin (phalloidin) and phosphopaxillin (*red*). *B*, MIN6B1 were cultured in 2.8 mm glucose for 4 h in the presence or absence (*CTL*) of PF562271 (*PF56*, 0.1 μm) and Rho activator (RhoAc, 0.25 µg/ml) and then stimulated or not for 20 min with 16.7 mm glucose. The cells were subsequently fixed and stained for actin (phalloidin in *cyan*) and paxillin (*green*). *C* and *D*, AKT2 isoforms or scramble (control) specific siRNA was transfected in rat primary β-cells for 72 h. The cells were cultured in 2.8 mm glucose for 4 h in the presence or absence of Rho activator (*RhoAc*, 0.25 µg/ml). *C*, the cells were then subsequently fixed and stained for actin to observe cell perimeter. Quantification of cell area was done using ImageJ software (*n* = 3). *D*, the cells were subsequently fixed and stained for actin (phalloidin in *cyan*) and paxillin (*green*). All images are fully representative of three independent experiments. *Scale bars*, 10 μm.

ROCK activation by RhoA has been involved in β-cell function and insulin secretion. For instance, pharmacological inhibition of RhoA and ROCK leads to a significant increase in actin depolymerization and GSIS in MIN6B1 and rat primary β-cells (30, 49), suggesting that through activation of this pathway, part of the insulin granule pool is prevented from reaching the plasma membrane (50). Further evidence supports a positive regulatory role for Rac1 in GSIS through F-actin reorganization (32). To determine whether these pathways regulate FA remodeling in β-cells, MIN6B1 cells were treated with specific inhibitors against ROCK (Y37632) and Rac1 (Rac1 inhibitor), and subsequent FA remodeling under low-glucose conditions was analyzed. Interestingly ROCK inhibition induced an increase of phosphopaxillin-containing adhesions, creating a phenotype similar to the glucose-stimulated condition (Fig. 8*A*). This suggested that the RhoA–ROCK pathway is necessary to maintain actin stress fibers, the large FA phenotype, as well as low levels of basal insulin secretion. In contrast, Rac1 inhibition did not modify the morphology of MIN6B1 cells under low-glucose conditions (Fig. 8*A*). To establish the hierarchy between FAK and RhoA signaling under low-glucose conditions, the RhoA–ROCK pathway was activated (RhoA activator, RhoAc) in the concomitant presence of the FAK/Pyk2 inhibitor PF562271. Although PF562271 treatment alone induced FA remodeling in MIN6B1 cells under low-glucose conditions, this remodeling was prevented by the simultaneous activation of RhoA (Fig. 8*B*). These results were confirmed in rat primary β-cells (Fig. S4), showing that FAK-dependent Rho–ROCK activation is critical to maintain stress fibers, large FAs, and the restriction of insulin granule release from β-cells under low-glucose conditions.

**Figure 9:**
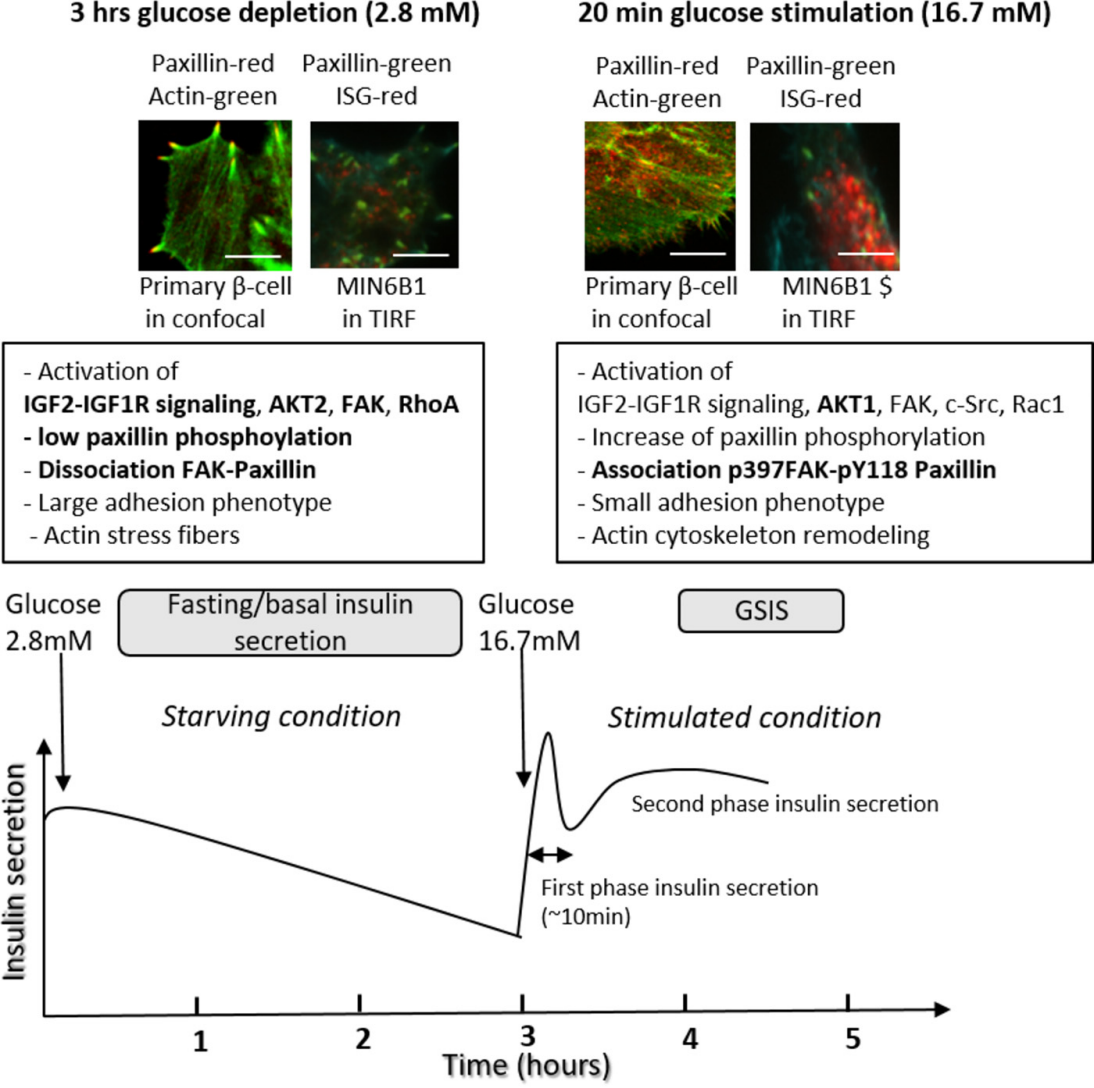
**Working hypothesis.** Once β-cells in the pancreas are switched to starving conditions in which glucose levels are low, their insulin secretion gradually declines. In response to an increase in glucose in the environment, GSIS is induced and proceeds in two phases that are either controlled by glucose metabolization in the mitochondria (first phase) or a second phase in which the morphology and cell–matrix adhesions of the β-cells are remodeled. For both the starvation and glucose-stimulated phases, the autocrine insulin/IGF2 signaling is critical but results in a different cellular response in respect to the specific glucose levels in the medium. During the second phase of GSIS (stimulated condition), the adhesion proteins paxillin, FAK, and c-Src are required for glucose-induced remodeling of the integrin-dependent adhesions and stimulation of Rac1 activity leading to F-actin remodeling. The adhesion and F-actin remodeling permits the trafficking of insulin secretory granule (*ISG*) toward basal membranes and subsequent exocytosis. This glucose-mediated mechanism is partially IR/IGF1R–AKT1–dependent. In contrast, under low-glucose conditions (fasting phase, starving conditions), IGF2 autocrine binding to the IGF1 receptor and downstream AKT2 activation led to the reinforcement of an integrin-dependent FAK-kinase, RhoA/ROCK signaling loop that maintains stress fibers and large focal adhesions to prevent inappropriate insulin release. Paxillin occupies a central position in this circuit, being therefore the potential glucose and AKT1/2 target to switch between the recruitment of FAK–RhoA to induce the large adhesion phenotype or Rac1, which induces the small adhesion phenotype, to control adhesion, cytoskeleton, and insulin release (see “Discussion”). *Scale bars*, 10 μm.

Finally, to determine the hierarchies between FA-mediated Rho–ROCK activation and the IGF1R–AKT2 signaling pathway, rat primary β-cells were transfected with siRNA against AKT2 (or scrambled control) and treated with RhoAc in low-glucose conditions. Importantly, activation of RhoA, even in the absence of AKT2, rescued the stress fiber and large FA phenotype, thereby preventing inappropriate cell spreading (Fig. 8*C*) and FA remodeling (Fig. 8*D*). These results propose that, on the one hand, IGF1R–AKT2 activation reinforces FAK-mediated signaling in FAs to maintain an elevated level of RhoA–ROCK activity under low-glucose conditions. On the other hand, a shift to high glucose stimulates an AKT1-mediated activation of the c-Src/p-Tyr-paxillin/FAK kinase activity, driving Rac1-mediated cytoskeletal and adhesion remodeling, leading to insulin granule release during the second phase of GSIS (see our working hypothesis; Fig. 9).

## Discussion

Regulation of insulin secretion by the pancreatic β-cell is key to normal glucose homeostasis. Within minutes after exposure to elevated glucose in the blood (corresponding to the post prandial condition *in vivo*), a rapid increase of cytoplasmic ATP leads to the opening of voltage-dependent calcium channels and subsequent intracellular calcium influx, which triggers a first rapid phase of insulin granule exocytosis (51, 52). A subsequent pool of granules is released during the second phase, which has been associated with the metabolization of alternative carbon sources, such as glutamine, and changes in integrin-dependent adhesions and cytoskeletal organization (22, 24, 53) and our own observations (Fig. 9). Under fasting conditions, β-cells strongly reduce the secretion of insulin granules to much lower but still detectable levels, also known as basal insulin secretion. Although an abnormal increased basal level of insulin secretion has been linked to the diabetic condition (1, 2), the mechanisms controlling this are still not yet elucidated (54, 55).

The present study confirms that IGF2 is released to signal in an autocrine fashion, both in the glucose-stimulated condition, to amplify the releasing signal (16), and under fasting conditions to maintain low basal insulin secretion in β-cells. We show here that an intricate link between the IGF2-mediated IR/IGF1R and integrin-dependent adhesions exists that can control the level of insulin granule secretion in a glucose- and metabolism-dependent manner. A glucose-dependent bifurcation of the IR/IGF1R downstream signaling pathway occurred at the level of AKT1 and AKT2 isoforms, which induce different β-cell morphologies and different signaling within integrin-mediated adhesions. They regulate RhoA/ROCK activity and thereby actin stress fibers and FA anchorage, leading to either an increase or a block in insulin granule release. Our results are the first evidence of an active role of IGF2–IGF1R–PI3K–AKT2–FAK–paxillin–RhoA–ROCK signaling, for stimulating a contractile phenotype, to prevent excessive insulin granule release under low glucose. In the presence of glucose, however, IR/IGF1R/AKT1 signaling participates in a FAK–c-Src–paxillin–mediated stimulation of Rac1 (56), leading to FA remodeling and cell spreading to increase insulin secretion in a glucose- and metabolism-dependent fashion (Fig. 9).

A debate is still ongoing concerning the autocrine action of insulin on β-cells. Indeed, several studies or animal models questioned the concept, whereas others confirmed it (7). In our study, we observed that antibody-mediated blockade of IGF2 in the medium, pharmacological inhibition of IR/IGF1R signaling, or knockdown of AKT isoforms showed defects in the regulation of insulin secretion in primary β-cells, validating the autocrine concept. Interestingly, despite the common autocrine function, different components of the pathway (*e.g.* AKT1 *versus* AKT2) may act in specific ways. IGF1R, but not IR, was for example shown to induce metabolic genes expression, which regulated PDX1 transcription and insulin expression (57). In the presence of high glucose, IR inhibition by S961 had a smaller negative effect on GSIS than inhibition of both IR and IGF1R by AG1024 (Fig. 1), suggesting that both signalings are necessary to fully enhance GSIS. However, exogenous insulin in the presence of low glucose (2.8 mm) did not have any direct effect on adhesion remodeling (data not shown), proposing that without extra glucose, additional insulin degranulation is not stimulated by released insulin. On the contrary and based on the AG1024 inhibition effects (Fig. 1, *C–E*), a low but persistent level of IGF1R signaling, but not IR signaling (Fig. 1*B*), restricts insulin secretion to basal levels, as well as maintaining RhoA activity, stress fibers, and FA adhesions (Fig. 8). According to our results, during prolonged periods of starvation, a reduction of IGF1R signaling would cause an increase in insulin granule release, which is observed in the physiopathology of diabetes (1). Cooperative signaling between IGF1R and integrin-mediated adhesion is essential for growth and migration of normal and cancer cells (58), and FAK-Akt was shown to promote pancreatic β-cell survival (59), suggesting that simultaneous activation of these two pathways is also required for the normal homeostasis of β-cell functions.

Interestingly, IGF2, which can bind to IR or IGF-IR/IR hybrid receptors (60), was shown to be secreted concomitantly with insulin and has been considered an important player in the autocrine regulation of β-cell mass and GSIS through IGF1R signaling (16, 17). Although insulin-mediated effects are very rapid, changes caused by altered IGF2-IGF1R signaling, such as FA remodeling or insulin secretion, were only apparent after 4 h of anti-IGF2 blocking antibody treatment (Fig. 3). Similarly, blocking FAK signaling, known to be relevant for cell survival, also resulted in adhesion remodeling after 4 h. In addition, p-ERK levels decrease only slowly after glucose starvation (3 h) in β-cells (our own observations), suggesting that significant changes in cellular signaling are not very rapid, potentially explaining that the observed low glucose–mediated effects were only relevant after a 4-h period. Alternatively to a slow decline of intracellular signaling, it is also possible that critical survival promoting factors, such as integrin-dependent adhesion to extracellular matrix cannot be blocked rapidly. Furthermore, it is conceivable that released IGF2 is maintained in the local microenvironment of β-cells, for example by binding to IGFBP proteins. IGFBP proteins are anchored to ECM components in the tissue and mediate IGF2 availability (61). For instance IGF2–IGFBP complexes have been found accumulated in pancreatic cancer (62). A failure to support β-cell survival and growth may be induced during inflammation or chronic hyperglycemia, when the pancreatic ECM and thus the tissue retention of IGF2–IGFBP complexes is altered. This scenario might cause progressive β-cell dysfunction, such as enhanced basal insulin secretion, which predispose to diabetes (63, 64). Still, further studies are required to understand the fate and tissue function of β-cell–secreted IGF2 in the pancreas.

Isoforms of the serine/threonine protein kinase AKT (1/2) are major downstream effectors of the IGF1R/IR and are known to play a central role in the modulation of β-cell function, proliferation, and apoptosis (65). Despite an ∼80% identity at the amino acid level, their functions, subcellular localization, and targets are distinct (40). For example, in skeletal muscle, AKT1 silencing affected the lipid metabolism, whereas AKT2 modified the glucose metabolism (41). Furthermore, it has been shown that AKT1 knockout mice had increased islet mass (66). In addition, the AKT downstream effector, Rab GTPase AS160, described to be involved in basal insulin secretion and GSIS (9), was specifically affected by targeting the AKT2 isoform in adipocytes. Here, we show an isoform specificity of AKT in the regulation of insulin secretion in β-cells. Although AKT1 is involved in GSIS, with only partial effects on F-actin and adhesion remodeling in high-glucose conditions (Fig. 4, *B*, *C*, and *E*), AKT2 stimulates the tensioned state of β-cells, by maintaining stress fibers and forming integrin-dependent anchorage, to prevent inappropriate insulin secretion under low-glucose conditions (Fig. 4, *B–E*). This indicates that the glucose-mediated switch or bifurcation of the IR/IGF1R signaling occurs at the AKT1/AKT2 level, suggesting that either a critical target or their activation is affected by the glucose metabolism and autocrine IGF2 signaling. However, how AKT isoforms regulate adhesion and actin cytoskeleton in general and in β-cell specifically is still unresolved.

Interestingly, AKT2 has been reported to inhibit migration through a block of Rac/Pak signaling in mouse embryo fibroblasts (67). In our hands, reactivation of RhoA signaling restored the normal phenotype in the AKT2 knockdown cells (Fig. 8, *C* and *D*). Thus, we expect that in low-glucose conditions, RhoA activation by AKT2 inhibits insulin granule exocytosis by blocking Rac1-dependent F-actin and FA remodeling. Accordingly, the inhibition of the RhoA-effector ROCK increased basal insulin secretion (30) and stimulated phosphopaxillin staining in FAs in low glucose (Fig. 8*B*), suggesting that actin cytoskeleton regulation through RhoA–ROCK signaling occurs downstream of AKT2 and FA/FAK activity (Fig. 8). Thus, a positive feedback between RhoA–ROCK-mediated F-actin tension, and FA-dependent FAK activation is operating under low glucose. When this feedback is disrupted by FAK kinase inhibition, F-actin and FA remodeling is induced, leading to enhanced basal insulin release.

The regulation of insulin release by AKT may directly occur at the adhesion level. Indeed, direct binding between AKT and FAK was previously shown in cancer cells (68). Knockdown of the AKT2 target AS160 decreased GSIS but increased basal insulin secretion in β-cell (9, 69), similar to what was observed in Figs. 4*A* and 5 (*C* and *D*) when AKT2 was knocked down or FAK/Pyk2 was inhibited. Interestingly, FAK regulates glucose-induced AS160 phosphorylation in β-cells (23), suggesting that the Rab GTPase activator (AS160) acts also downstream of FA-associated FAK signaling. Thus, it is conceivable that both AKT2 and FAK target AS160 to control basal insulin secretion, strongly suggesting that under fasting conditions, both IGF2-IGF1R–AKT2-AS160 and FAK-RhoA/ROCK signaling are required to maintain basal insulin secretion at physiological levels.

The FA genesis maturation and their regulation in β-cells are still unknown. However, our observations suggest important roles of the FA-associated proteins FAK/Pyk2, paxillin, and SFK in the control of insulin secretion. Consistent with previous reports and confirmed in β-cell in the present study, glucose induces the phosphorylation on Tyr^397^ of FAK (22, 23), leading to the recruitment of c-Src (27), which in turn will phosphorylate the Tyr^118^ of paxillin in nascent adhesion (36, 45), crucial mechanisms to fully enhance insulin secretion. Indeed, FAK/Pyk2 kinase inhibition induced F-actin and FA remodeling (Fig. 6*A*), associated with an increase of paxillin phosphorylation on Tyr^118^ (Fig. 7*B*), mediated by SFK (Fig. 6*B*), leading to an augmentation of basal insulin release (Fig. 5, *B* and *C*) adding complexity to the interaction between these components in a low-glucose scenario. Interestingly, in mammary epithelial cells, similar observations were made; reducing the stiffness of the ECM decreases FAK phosphorylation but increases the activation of c-Src (70). In the case of β-cells, this suggests that maintaining intracellular tension and FA-associated FAK activity restricts the activating signaling of the p-paxillin–c-Src complex to pre-existing FAs, thereby preventing the induction of cell spreading and nascent adhesion formation (36). If paxillin is maintained in the dephosphorylated state (Tyr^31/^Tyr^118^), as for example in the Y31F/Tyr^118^F mutant, stress fibers and elongated FAs are found (71) (Fig. 7*B*), suggesting that the regulation of paxillin phosphorylation and its association with FAK is critical to maintain low levels of insulin secretion (Fig. 2*B*).

How IGF1R signaling regulates adhesion proteins to maintain proper adhesion morphology and insulin secretion is still unclear. Recently, it has been demonstrated that the YES kinase, another SFK, is recruited at basal membrane of β-cell and involved in GSIS (29). This kinase could also be involved in the regulation of basal insulin secretion, especially because IGF1R inhibition has been shown to activate YES proteins in cancer cells (72). Unfortunately, because of experimental limitation (no antibody worked in our cells), we failed to demonstrate an involvement of YES in our context. Another candidate was the nonreceptor tyrosine kinase Csk, which serves as a negative regulator of the Src family tyrosine kinases by specifically phosphorylating the negative regulatory site of SFK (73). Recently, AKT was shown to target and activate Csk in germinal cells (74). In our context, activation of the IGF1R–AKT2 signaling could activate Csk, which in turn will suppress c-Src or YES activity and thus result in low levels of paxillin phosphorylation to maintain large FAs, to repress insulin secretion under fasting conditions. Further analysis is required to confirm this hypothesis because Csk has never been studied in β-cells.

To summarize, the context of β-cells offers a particular cellular system in which the constitutive uptake and efficient metabolic conversion of glucose is used to regulate the release of prestored insulin granules. Deleterious IR/IGF1R signaling or altered FA composition and function may lead to fasting hyperinsulinemia and a decrease of GSIS observed in the physiopathology of diabetes. *In vivo* studies support this hypothesis, because β-cell–specific knockout of IR, IGF1R (3, 4, 5), or AKT (6) induced fasting hyperinsulinemia and diabetes. In addition, emerging evidence suggests dysfunctions of integrin-mediated adhesions in diabetes that either affect regulated insulin release *in vitro* (50) or can be linked to altered actin remodeling and FAK phosphorylation in β-cells derived from diabetes type 2 patients.

## Experimental procedures

### Antibodies and reagents

The following antibodies were used: rabbit anti-Akt1 (catalog no. 2938), rabbit anti-Akt2 (catalog no. 3063), rabbit anti-ERK1/2 (catalog no. 9102), rabbit anti-p(Tyr^118^) paxillin (catalog no. 2541), rabbit anti-pS473 Akt (catalog no. 9271), rabbit anti-Akt (catalog no. 9272), and rabbit anti-Src (catalog no. 2108) from Cell Signaling Technology; mouse anti-paxillin (catalog no. 610051), mouse anti-actin (catalog no. MAB1501), and rabbit anti–pT185/Y187 ERK1/2 (catalog no. 92590) from Merck Millipore; rabbit anti-pTyr^397^ Fak (catalog no. 700255), rabbit anti-p(Tyr^118^) paxillin (catalog no. PA5-178028), rabbit anti-p-Tyr^418^ c-Src (catalog no. 44-660), Alexa Fluor 647–phalloidin to visualize F-actin (catalog no. A22287), anti-rabbit Alexa Fluor® 488 (catalog no. A21206), anti-rabbit Alexa Fluor® 555 (catalog no. A31572), anti-mouse Alexa Fluor® 555 (catalog no. A31570), and anti-mouse Alexa Fluor® 488 (catalog no. A11001) from Thermo Fisher Scientific–Invitrogen; rabbit anti-FAK (catalog no. sc-558) from Santa Cruz Biotechnology; donkey anti-rabbit horseradish peroxidase (catalog no. NA934V) and donkey anti-mouse horseradish peroxidase (catalog no. NA931V) from GE Healthcare; and rabbit anti-IGF2 (ab9574) from Abcam. The following reagents were used: PF562271 (catalog no. S2890) ATP-competitive FAK/Pyk2 inhibitor and saracatinib (catalog no. AZD0530) SFK inhibitor from Selleckchem; tyrphostin AG1024 (catalog no. ALX-270-217) and specific inhibitor of IGF-1 receptor from Enzo Life Science; Rac1 inhibitor (catalog no. 553502) and LY294002 (catalog no. 440202) PI3K inhibitor from Calbiochem and Merck Millipore; Y27632 (catalog no. 1254) ROCK inhibitor from Tocris Bioscience; Rho activator II (RhoAcII: catalog no. CN03) from Cytoskeleton, Inc.; and S961 specific insulin receptor antagonist (catalog no. 051-86) from Phoenix Pharmaceuticals.

### TUNEL assay

After treatment (Krebs–Ringer bicarbonate buffer (KRB) + glucose or inhibitors), the cells were fixed with 2% paraformaldehyde. The free 3-OH strand breaks were detected by the TUNEL technique according to the manufacturer's instructions (*in situ* cell death detection kit; Roche, Switzerland).

### RNAi-mediated knockdown of endogenous Akt1 and Akt2 by transient transfection

Knockdown of Akt1 and/or Akt2 in rat primary β-cells was achieved by transfection with specific siRNA (Akt1: SASI_Rn01-00063656, Akt2: SASI_Rn01_00047688, Sigma–Aldrich; and control siRNA (Signal Silence Control siRNA, Cell Signaling Technology, Danvers, MA). In brief, liposome-siRNA, Lipofectamine 2000 (Life Technologies, Inc.) and 100 nm siRNA was prepared in 200 μl of opti–modified Eagle's medium as described by the suppliers. Transfected primary β-cells were incubated 72 h to allow siRNA expression before cell treatment and analysis.

### MIN6B1 mouse pancreatic cell line

MIN6B1 cells were cultured as previously described (75) and plated on plastic dishes (protein study and confocal imaging) or 35-mm glass-bottomed dishes coated with 804G-ECM for TIRF microscopy (76).

### Islets and primary β-cell purification and culture

Islets of Langerhans were isolated by collagenase digestion of pancreas from adult male Wistar rats followed by Ficoll (Histopaque-1077; Sigma–Aldrich) purification based on a method adapted from the work of Rouiller *et al.* (77). The islets were digested with trypsin-EDTA (0.02%; Life Technologies, Inc.). β-Cells were separated from non–β-cells by auto-FACS using a FACS (Bio-Rad). Sorted rat β-cells were cultured in Dulbecco's modified Eagle's medium containing 11.2 mm glucose, 0.05 mg/ml gentamicin, 100 units/ml penicillin, 100 mg/ml streptomycin, and 10% fetal calf serum (Sigma–Aldrich). Sorted rat β-cells were cultured on plastic dishes coated with extracellular matrix derived from 804G cells as described elsewhere (78) (produced by us) and were left for 24 h to adhere and spread before initiation of the experiments. Animal experimentation authorization number GE/144/18 was provided by the Swiss Cantonal Veterinary Office.

### Insulin secretion

β-Cells or islets were washed, preincubated, and incubated for insulin secretion assays, and insulin was measured by radioimmunoassay as described in Ref. 79 or by rat (catalog no. 10-1250-10) or mouse (catalog no. 10-1247-10) ELISA as according to the manufacturer's instructions (Mercodia). In brief, the cells were preincubated 2 h in KRB at 2.8 mm glucose followed by a further 1 h or more (as indicated) at 2.8 mm to measure basal insulin secretion and a further 20 min or 1 h (as indicated) at 16.7 mm glucose to measure stimulated secretion. Inhibitors were added to the incubations as indicated for each specific experimental condition. Cellular insulin was extracted at the end of the experimental period, and secretion was expressed as a function of total insulin (cell content + basal + stimulated secretion).

### Expression vectors

The plasmid expressing NPY–Cherry was a gift from G. Rutter (Imperial College London, London, UK), paxillin–pEGFP was from AddGene (Cambridge, MA, USA), paxillin–Cherry mutated for Y31F and Y118F was produced in our laboratory by Marta Ripamonti. These plasmids (1 μg) were transfected in MIN6B1 cells plated on glass-bottomed dishes coated with ECM from 804G cells for 72 h using Lipofectamine 2000 according to the manufacturer's instructions.

### TIRF microscopy

Transfected MIN6B1 cells were incubated in KRB in presence or absence with the corresponding inhibitor. TIRF images were obtained as earlier described (23).

### Immunofluorescence

Basal membranes or the central plane of cells were observed by confocal microscopy using a Zeiss LSM700 Meta microscope with a 63× oil immersion lens, and images were acquired and processed using ImageJ (National Institutes of Health). *Scale bars*, 10 μm.

### Co-immunoprecipitation

Sorted rat primary β-cells (minimum of 100,000 cells/conditions) coated on 804G were incubated in KRB (specific condition specified in the figure legend), and then cell lysates and 30–50 μg of protein were incubated with 5 μl of FAK antibody for 1 h at 4 °C. Next, lysate + antibody were added to 20 μl of protein A/G–agarose (catalog no. sc-2003, Santa Cruz) and incubated overnight at 4 °C. After centrifugation, the supernatants were collected, and then beads were washed three times with lysis buffer at 4 °C. Laemmli buffer was added on beads to elute FAK and paxillin.

### SDS-PAGE and Western blotting

Protein samples were prepared and immunoblots analyzed as described (79). Western blots were quantified by densitometry, and band density of phosphoproteins was normalized to that of the corresponding total protein and/or to total actin as indicated in the figure legends.

### Statistical analysis

Statistical significance for differences between experimental conditions was determined using GraphPad Prim 5 software by one-way analysis of variance (for all experiments which compared more than two conditions) or two-way analysis of variance (for insulin secretion experiments that compared basal and stimulated conditions) with Tukey post hoc test for multiple comparison analysis or by Student's *t* test (when only two conditions were compared). The data are means ± S.E., independent experiments, and *P* values less than 0.05 were considered significant (*ns*, nonsignificant; *, *p* < 0.05; **, *p* < 0.01; ***, *p* < 0.001).

## Data availability

All data are contained within the article and supporting information.

## Footnotes

3 C. Arous, K. Rickenbach, and B. Wehrle-Haller, unpublished observations.
