# Supplementary material for: Integrin and autocrine IGF2 pathways control fasting insulin secretion in β-cells
Source: J Biol Chem. 2021 Jan 13;295(49):16510–28. doi: 10.1074/jbc.RA120.012957 (PMC7864053; doi:10.1074/jbc.RA120.012957)
Supplement: Supplementary file 1 [file mmc1.pdf]

Integrin and autocrine IGF2-pathways control fasting insulin secretion in β-cells

Caroline Arous[1], Maria Luisa Mizgier[2], Katharina Rickenbach[1], Michel Pinget[2], Karim Bouzakri[2*] and Bernhard Wehrle-Haller[1*]

From the [1] Department of Cell Physiology and Metabolism, Centre Médical Universitaire, University of Geneva, Switzerland
[2]UMR DIATHEC, EA 7294, Centre Européen d'Etude du Diabète, Université de Strasbourg, France

Running title : Integrin and IGF2-pathways control fasting insulin secretion

S-1

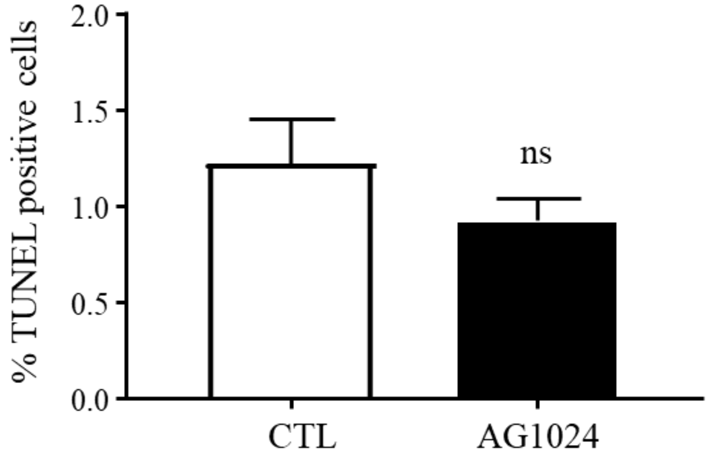

Supp info 1: **AG1024 did not induced cell death in low glucose condition**. Rat primary β-cells were cultured in 2.8 mM glucose medium for 5h, containing none (CTL) or AG1024. After cell fixation, TUNEL assay were performed according to the commercial instruction.

S-2

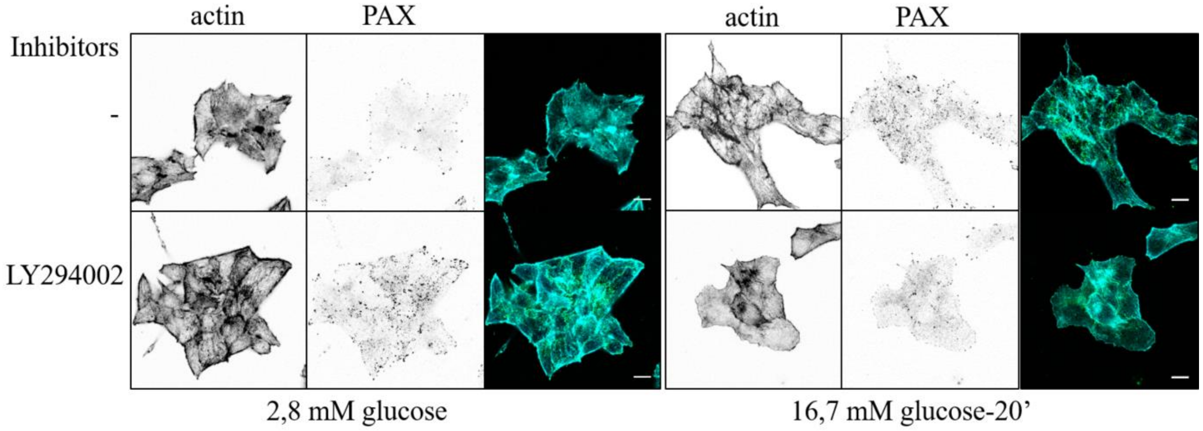

Supp Info 2: Inhibition of PI3K activity induced remodeling in low glucose condition but blunted glucose-induced remodeling in MIN6B1 cell. MIN6B1 were cultured in 2.8 mM glucose for 4h in presence of LY294002 (10 µM) or not, and then stimulated or not for 20 min with 16.7 mM glucose. Cell were subsequently fixed and stained for actin (phalloidin in cyan) and paxillin (green). All images are fully representative of 3 independent experiments. Scale bar: 10µm.

S-3

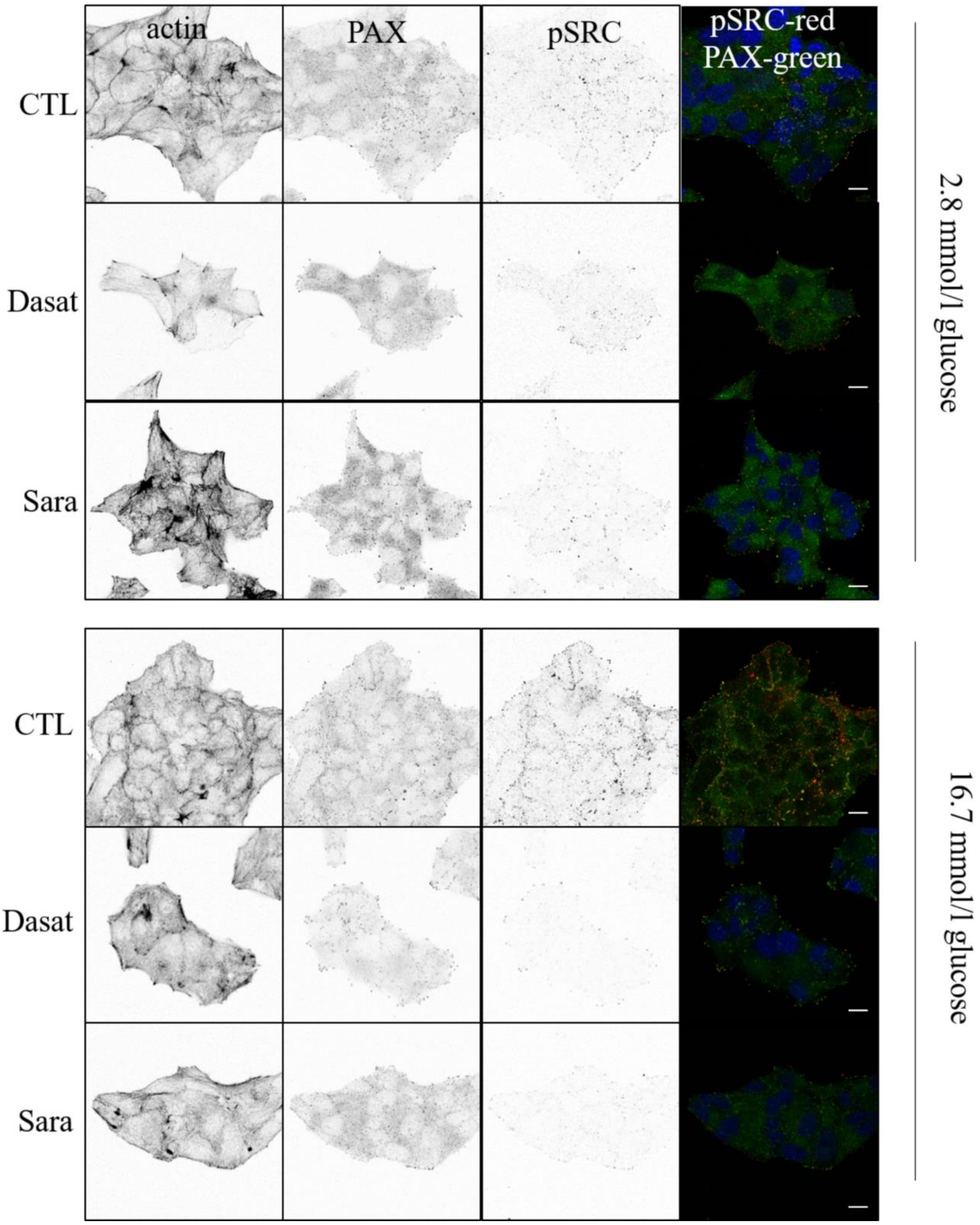

Supp info 3: Dasatinib and saracatinib effect on phospho-c-Src and adhesion remodeling. MIN6B1 cells were cultured in 2.8 mM glucose for 3h in presence or not (CTL) of dasatinib (dasat) or saracatinib (sara) and then stimulated or not with 16.7 mM glucose for 20 min in the continued presence of the inhibitors. Cell were subsequently fixed and stained for actin (phalloidin), paxillin (green) and phospho (Y418) c-Src (red). All images are fully representative of 3 independent experiments. Scale bar: 10µm.

S-4

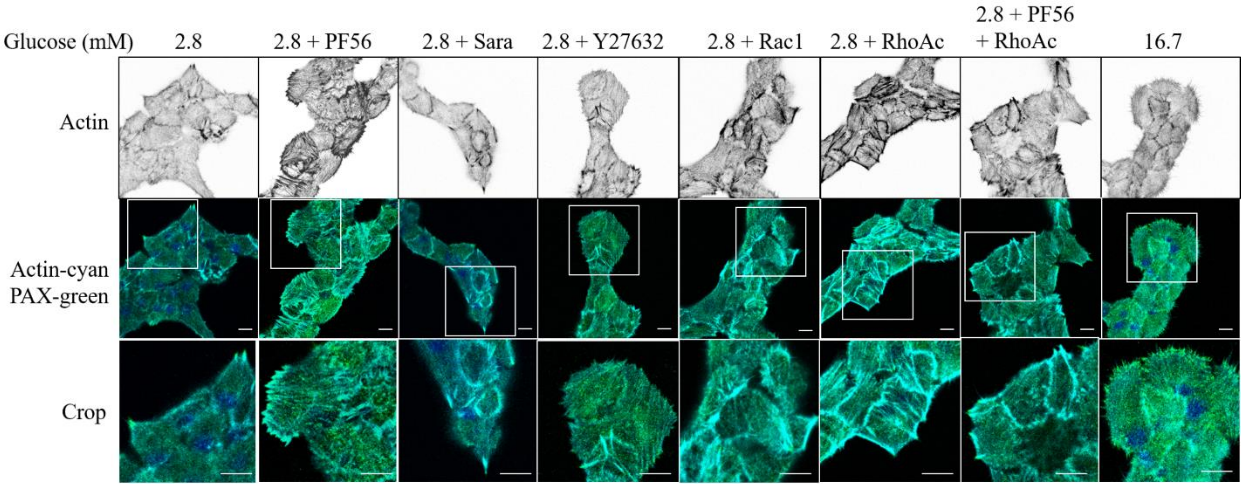

Supp info 4: Effect of inhibitors on adhesion remodeling in rat primary β-cell. Rat primary β-cells were cultured in 2.8 mM glucose for 4h in presence or not (CTL) of inhibitors and then stimulated or not for 20 min with 16.7 mM glucose. PF562271 (PF56: 0.1 µM), Saracatinib (Sara:0.1 µM), ROCK inhibitor (Y27632: 50 µM), Rac1 inhibitor (Rac1: 0.2 µM) and Rho activator (RhoAc: 0.25 µg/ml). Cell were subsequently fixed and stained for actin (phalloidin) and paxillin (green). All images are fully representative of 3 independent experiments. Scale bar: 10µm.
